# HIV-1 Non-Group M Strains and ART

**DOI:** 10.3390/v15030780

**Published:** 2023-03-17

**Authors:** Elodie Alessandri-Gradt, Alice Moisan, Jean-Christophe Plantier

**Affiliations:** Univ Rouen Normandie, UNICAEN, INSERM, DYNAMICURE UMR 1311, and CHU Rouen, Department of Virology, National Reference Center of HIV, F-76000 Rouen, France; elodie.alessandri@chu-rouen.fr (E.A.-G.); alice.moisan@chu-rouen.fr (A.M.)

**Keywords:** HIV-1, genetic variants, ART, elimination

## Abstract

To eliminate HIV infection, there are several elements to take into account to limit transmission and break viral replication, such as epidemiological, preventive or therapeutic management. The UNAIDS goals of screening, treatment and efficacy should allow for this elimination if properly followed. For some infections, the difficulty is linked to the strong genetic divergence of the viruses, which can impact the virological and therapeutic management of patients. To completely eliminate HIV by 2030, we must therefore also be able to act on these atypical variants (HIV-1 non-group M) which are distinct from the group M pandemic viruses. While this diversity has had an impact on the efficacy of antiretroviral treatment in the past, recent data show that there is real hope of eliminating these forms, while maintaining vigilance and constant surveillance, so as not to allow more divergent and resistant forms to emerge. The aim of this work is therefore to share an update on the current knowledge on epidemiology, diagnosis and antiretroviral agent efficacy of HIV-1 non-M variants.

## 1. HIV-1 Non-M Variants

Human immunodeficiency virus (HIV) presents an important genetic diversity. The first HIV was isolated at the Pasteur Institute of Paris in 1983 [1]. The existence and circulation of other variants, genetically and/or antigenically different, have been demonstrated since 1985 by unusual serological profiles among Senegalese people [2]. In 1986, a new variant, harboring a strong genetic divergence compared to the first strain isolated, with over 50% sequence divergence in the envelope gene, led to the differentiation into HIV types 1 (HIV-1) and 2 (HIV-2) [3]. The identification of other major variants, with less marked but notable genetic divergences, has led to the definition of four groups of HIV-1. The first one was corresponding to that linked to the first strain discovered in 1983, which is now predominating in the HIV pandemic, and was designated HIV-1 group M (HIV-1/M) for “major”. The three other groups, all identified in people of Cameroonian origin, were classified as group O (HIV-1/O) for “outlier” in 1994 [4], group N (HIV-1/N) for “non-M, non-O” in 1998 [5] and group P (HIV-1/P), to follow the nomenclature, in 2009 [6].

**Figure 1:**
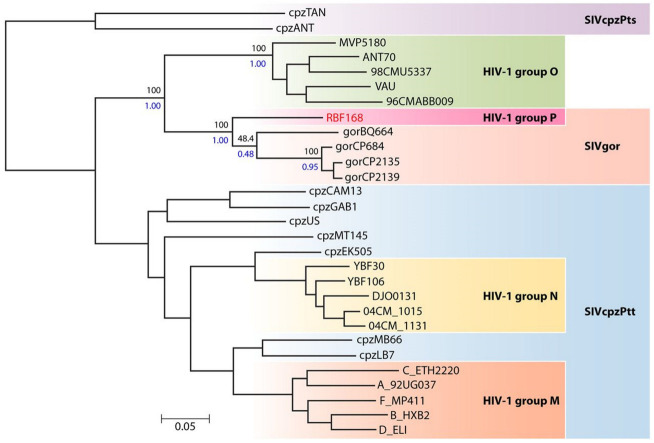
Phylogenetic relationships between the HIV-1, SIVcpz and SIVgor lineages (reprinted from reference [6] with permission of the publisher).

Several factors account for this considerable genetic diversity. First, HIVs correspond to a zoonotic origin from viruses found in great apes. Studies in Cameroon have shown that distinct SIVcpz and SIVgor are endemic in wild chimpanzees and gorillas, respectively; therefore, HIV-1/M and HIV-1/N arose from independent transmissions from troops of chimpanzees infected by distinct SIV variants, located in distinct regions [7,8] (Figure 1). HIV-1/O and HIV-1/P arose from independent transmissions of troops of gorillas [9] (Figure 1). After each successful transmission event between apes and humans, the different variants have evolved according to their own selection pressure and epidemiological factors, contributing strongly to the diversification of HIV over time.

The natural variability of HIV, characterized by a high rate of replication, a large daily production of viral particles and a low fidelity of its reverse transcriptase (RT), as well as the establishment of quasispecies, also explains the great genetic diversity of HIV [10,11,12].

At last, genetic recombination, consisting of the formation of a chimeric genome composed of several genomic fragments of different parental origin, also accentuates the diversification of the quasispecies by helping repair damage due to deleterious mutations [13] and influencing viral fitness [14], immune escape [15] and resistance emergence [16,17]. Molecular epidemiology studies have highlighted the importance of recombination in generating viral diversity throughout the current pandemic [18]. Indeed, intra-group M recombination corresponds to 18% of HIV infections worldwide [19], proving that genetic recombination contributes to the great genetic diversity of HIV. Given the significant genetic divergences between HIV-1/M and HIV-1/O and the low prevalence of HIV-1/O, recombination between these two groups has been considered negligible and has long been very little investigated. It was not until 1999 that the first two recombinant forms were described [20,21].

## 2. Epidemiology

**Figure 2:**
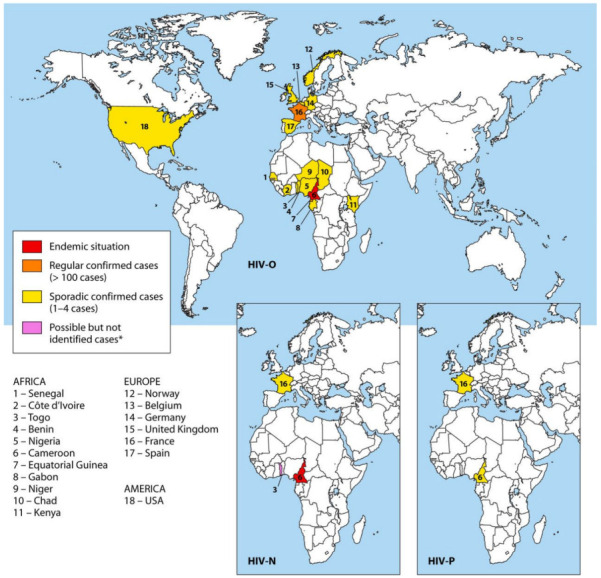
Molecular epidemiology of HIV-1 non-group M. The three maps represent the worldwide distribution of the HIV-1/O, HIV-1/N and HIV-1/P variants, reprinted from [22]. * One case of group N infection was detected in France but has likely its origin in Togo [23].

HIV-1 non-M strains are mainly present in Cameroon and in neighboring countries; this endemicity suggests that Western Central Africa is the source of the different HIV-1 groups (Figure 2).

HIV-1/O infections were described in different countries of Western Central Africa [24,25,26,27]. However, it is in Cameroon that HIV-1 group O is mainly found, with 0,6 to 1% of all HIV infections in this country [28]. Co-circulation of groups M and O in Benin and in Cameroon has led to the description of 16 cases of dual M and O (HIV-1/M + O) infections, associated or not with HIV-1/MO recombinant forms [29,30,31,32]. The first HIV-1/MO recombinant virus was reported from an asymptomatic Cameroonian woman, in 1999 [20]. Since then, 11 HIV-1/MO recombinants have been described in Cameroon [21,29,31,32].

By now, only 22 reports of HIV-1/N infection have been described, all but one in Cameroon [5,22,33,34,35,36,37,38,39,40].

Two cases of HIV-1/P infection have been reported to date in patients of Cameroonian origin [41,42].

HIV-1/O infection is also sporadic outside Western Central Africa and has been reported in West and East Africa [43,44,45,46,47,48], the United States and Europe [4,49,50,51,52,53] but always connected to patients or partners of patients originating in Cameroon (Figure 2). The RES-O (a French network) has been set up by the National Reference Center of HIV in France, with the aim of monitoring the spread of these variants and characterizing them. Since the first case described in 1992, one hundred forty-four HIV-1/O-infected patients have been described [54,55]. Moreover, two cases of HIV-1/M + O dual infection and several HIV-1/MO recombinants have also been detected outside Western Central Africa since 2004 and 2010, respectively. To date, 5 and 12 cases of HIV-1/M + O dual infections and of HIV-1/M + O recombinant forms, respectively, have been described, in several studies, all in France [56,57,58,59,60]. All these cases were identified in patients with an epidemiological link with Cameroon. Overall, since the description of the first case in 1999 in Cameroon, 25 HIV-1/MO recombinants have been identified in 24 patients, grouped into 20 URF_MO [61] (NRC data, manuscript in preparation).

Regarding HIV-1/N, one case was described outside Cameroon [23], with diagnosis in France of primary HIV infection in a Togolese patient.

With the discovery of the HIV-1/P prototype strain in France, these data show that there is no border for the circulation of non-M variants and that regular surveillance of genetic diversity is needed in Western Central Africa and abroad.

## 3. Diagnosis and Virological Monitoring

Group N and P viruses do not lead to difficulties for serological diagnosis [22]. The improvement of enzyme-linked immunosorbent assays has reduced the risk of failure to detect group O infection; however, some diagnosis failures have been reported, especially with rapid diagnosis tests (RDTs) or tests that do not include group O specific antigen [62,63,64,65,66].

It is therefore necessary to remain vigilant when facing clinical situations suggestive of HIV infection and negative HIV serology results (especially when using RDT or when diagnosing primary infection, in situations of undetectable viral load (VL) in the absence of treatment, or immuno-virological dissociation). Moreover, the absence of discrimination among HIV groups (i.e., to give a result variant-specific as for HIV-2), in the endemic region may lead to an underestimation of the number of these infections.

The genetic diversity of group O variants had a significant impact on the first commercial kits for quantifying their plasma RNA [67]. The development of non-specific kits that can quantify group M and O strains has improved the monitoring of these patients and more largely of infections by non-M variants [6,68,69,70,71,72]. The plasma viral load (pVL) can now be assessed using several commercial tests from Abbott, Altona, Cepheid, Hologi and Roche. Their reliability is correct, even if discrepancies exist, sometimes significant, as with the Hologic kit for O variants or Abbott for N variants [72,73,74,75] (NRC data, manuscript in preparation).

## 4. Antiretroviral Agents

The high genetic diversity of non-M variants has consequences for antiretroviral treatment (ART) management, which has not been well described until recently. Indeed, during the pre-clinical development and the approval process of antiretroviral drugs, only a very few strains of HIV-1/O are usually tested in vitro, not reflecting the whole diversity of these HIV-1 groups. Due to the restricted circulation of HIV-1/O strains and scarcity of the other non-M groups, the clinical studies could not enroll such infected patients. So to date, there is still no HIV-1 non-M algorithm for the interpretation of genotypic resistance. The use of current algorithms, defined for HIV-1/M, can only be suggestive and should be carefully interpreted [76].

### 4.1. Natural Resistance (In Vitro Data)

To date, there is no specific natural genetic polymorphism associated with HIV-1/N strains [33,35,77]. However, due to the very limited data available for this group, careful attention is still required.

The whole genome sequences of the RBF168 prototype strain and U14788, the second P-group strain discovered so far, are the only genotypic data we currently have about HIV-1/P natural polymorphism [6,42]. These two strains are very close genetically, but some differences can persist in the genes associated with the therapeutic region [41]: for example, these strains shared a high natural polymorphism in the protease region (12 mutations) and five mutations in the RT and the L44M in the Gp41 region. However, RBF168 had specific I15V in the protease region, whereas U14788 specifically harbored K101Q and E138D in the RT region and T97A in the integrase.

Concerning HIV-1 group O, the natural genetic polymorphism is now well determined by comparison with HIV-1 group M [76]. The main issue since 1997 was the natural resistance associated with non-nucleoside RT inhibitors (NNRTIs) [78,79,80,81] through the well-known Y181C. This mutation was present in 54 to 79% of the strains, depending on the studies [76,82], and is likely to be subgroup-dependent (H or T) [83,84]. However, the second generation of NNRTIs, including first etravirine and then doravirine, could have inhibitory activity on HIV-1 group O due to a different profile of resistance. This hypothesis has already been demonstrated for etravirine. Indeed, its phenotypic activity on group O strains was less affected (fold change (FC) of 10) than that of nevirapine (FC of 89) or efavirenz (FC of 42) [79]. It has also been demonstrated that additional mutations on the RT region could decrease drug susceptibility. For example, K103R, associated with natural A98G and V179E, had a 10-fold increase effect on etravirine and nevirapine susceptibility [78,79]. However, for now, doravirine which is the most recent NNRTI drug, seems to have a singular genotypic profile that could overcome the previous barrier to resistance. According to the current predictive algorithms, Y181C alone does not lead to doravirine genotypic resistance, but doravirine genotypic resistance appears if Y181C is co-expressed with K103N. Phenotypic studies and in vivo data are still missing to determine the full efficacy of doravirine in treating HIV-1/O infection.

Associated with this main mutation, all the group O strains shared the mutations A98G, V118C, V179E and L210Y on the RT region [76]. The impact of the atypical mutation L210Y (the 210W is a resistance residue) on nucleos(t)ide RT inhibitor (NRTI) resistance is still not known, but it could be minor [79]. A phenotypic study, conducted on 18 group O primary isolates and 1 MO recombinant, by comparison to group M strains, indicated similar IC50s for lamivudine between group M and O strains (*p* = 0.145) [79]. Only one isolate expressed a phenotypic resistance with a 125-fold higher IC50 than the mean IC50 (0.05 nM). Actually, this isolate, harbouring the M184V resistance mutation, was derived from a patient in whom an NRTI dual-therapy of zidovudine + lamivudine previously failed.

With more than 34% of the genome positions affected by a mutation, the protease region showed higher genetic polymorphism compared to the other regions. Some of these mutations are constantly present in group O strains (I13A, K20C, I62V and I93L for example) [76,85,86]. Consequently, the most frequent genotypic profile of natural mutations of resistance in the protease region is as follows: 10V + 15V + 20C + 36I + 58E + 62V + 63T + 69R + 71V + 89I. According to the main genotypic resistance algorithms (Stanford, ANRS, Rega), this profile implies a full susceptibility to protease inhibitors (PIs) except for atazanavir (possible resistance with ANRS interpretation), depending on the algorithm. The phenotypic impact of this polymorphism on PI susceptibility had not been well studied. An old study conducted in vitro on eight clinical HIV-1/O isolates demonstrated various ranges of susceptibility to saquinavir compared to group M, but without statistical confirmation [78].

The Gp41 region of group O strains is characterized by the well-conserved N42D mutation, associated with resistance to the fusion inhibitor enfuvirtide. This mutation was detected in 99% of the strains [80]. Other atypical mutations (N42NS, N43K, L45LP) were detected at a low level of <0.5%. Atypical mutations mean that the residues identified are not known for their association with resistance but are present in a position affected by resistance (for example N42S, position 42, is known for resistance with 42D residue, but not with S residue). Despite this theoretical genotypic resistance, Depatureaux et al. demonstrated in an in vitro phenotypic assay that 29 group O primary clinical isolates displayed IC50 susceptibilities from 4 to 5 000 nM, similar to those of group M [87]. These results confirmed that genotypic predictions from the algorithms of rules designed for group M are probably not completely suitable for group O antiretroviral susceptibility prediction [76].

The Gp120 region of HIV-1/O is known to have a much higher genetic diversity than HIV-1/M [88,89]. Thus, the tropism determination by the usual predictive genotypic algorithm designed for HIV-1-/M is not adapted for group O. Only one study reported a co-receptor determination assay in U87-CD4(+)CCR5 and CXCR4 cells infected by 18 HIV-1 O strains [79]. The results were also compared to the V3 sequences of each strain to test the sensitivity of current algorithms to predict co-receptor usage. More than 80% of the panel expressed CCR5 co-receptor usage and allowed testing maraviroc CCR5-antagonist activity. The others strains were dual-mixed (double use of CCR5 and CXCR4 co-receptors). Finally, none of the tested algorithms (PSSM, Geno2Pheno, net charge rule and 11/25 rule) were able to predict the group O tropism correctly. In this study, the R5 strains were all susceptible to maraviroc but with higher variations in IC50 ranges [min; max] than those for group M strains: [1;315 nM] and [2;102 nM], respectively. Although these results have to be confirmed in a larger panel and eventually with another phenotypic model, they indicated that maraviroc could be a therapeutic option for infection with an R5-tropic group O strain. In the same way, fostemsavir is a new attachment inhibitor preventing conformational changes in Gp120 and the CD4+ receptor on T-lymphocytes. To investigate in which way the high genetic natural polymorphism of non-M strains acts on this recent antiretroviral drug, a genotypic analysis on 111 sequences from HIV-1/O (n = 100), HIV-1/N (n = 9) and HIV-1/P (n = 2) was conducted [90]. From the eight substitutions associated with resistance (L116P, A204D, S375M/H, M426L, M434I, M475I and V506M) in group M, 100% of N strains harbored three mutations (S375M, M426L and M434I) corresponding to a fully genotypic resistance to fostemsavir. It was less clear for group O sequences, with 1% and 10% of the panel showing a dual mutation pattern, S375H + M426L and S375H + M434I, respectively, and group P sequences presenting multiple substitutions at resistance positions. Once more, extensive phenotypic studies are still required to clarify the susceptibility of group O to fostemsavir; so, this new drug should be used carefully, if used as a therapeutic alternative.

A natural genotypic polymorphism of the integrase region, harboring the 3 mutations L74I, A99S and T206S, was found at a high frequency (>99%) in several studies [82,91,92]. Due to the current recommendations to use, as first-line treatment, the integrase strand transfer inhibitors (INSTIs), it was necessary to investigate the impact of this natural polymorphism on the non-group M susceptibility to INSTIs. Thus, further phenotypic studies have been conducted on a large panel of around 40 clinical isolates, mainly HIV-1-/O isolates, for comparison of IC50 to susceptible HIV-1/M to obtain fold change (FC) values [93,94]. Most importantly, there was no intrinsic resistance to INSTIs for any isolate. For the five INSTI drugs tested, the median IC50s for HIV-1/O were 0.51 nM, 0.46 nM 0.30 nM, 1.59 nM and 4.59 nM for raltegravir, elvitegravir, dolutegravir, bictegravir and cabotegravir, respectively. All the HIV-1-/O strains were susceptible to INSTIs with FC < 2.5 except for elvitegravir; 15% of the panel had FC ≥ 10. This observation was linked to a low IC50 from the HIV-1/M and was consistent with previous results [79]. Comparing the drugs’ chemical structures, a higher heterogeneity of IC50 was found with elvitegravir compared to raltegravir. Interestingly, IC50 obtained with cabotegravir was also significantly different from that of bictegravir (*p* = 0.001) with a higher variability (as observed for group M isolates). It is still unclear if this finding is related to a specific genotypic pattern or a different activity in integrase enzymatic processing [95].

### 4.2. Antiretroviral Therapy and Virological Failure (In Vivo Data)

Studies on ART efficacy were extremely sparse and mostly old for HIV-1 non-M variants since no cohorts were available [85,92,96,97,98,99]. 

More recently, two studies on the largest group O-infected population have given significant data on ART efficacy in this group of variants, with current strategies of therapeutic management [100,101]. The first study analyzed data on immuno-virological responses to combination antiretroviral therapy (cART), in 80 patients monitored in France between 1996 and 2014, according to evolutive recommendations for HIV-1/M therapeutic management [100]. The cARTs initiated were mainly 2 NRTIs + 1 PI/r (51.3%), and 2 NRTIs + 1 NNRTI (18.8%). Data showed that ART-naive patients who started with a cART had a mean of +147 CD4+/μL after 12 months; a pVL < 200 cp/mL was found in median 3.1 months after cART initiation for 91.8%, and it took 3.8 months for 89.3% to reach < 40 cp/mL. Data also showed that around 20% of patients were treated with a non-recommended NNRTI-based regimen, which led to a gain of only 100 CD4+/μL after 1 year. For patients managed since 2007, the CD4+ count was at a median of 498 cells/μL, and 87% of them had an undetectable pVL (<40 cp/mL) at the last visit. This work showed that HIV-1/O-infected patients can be efficiently managed on the basis of the current HIV-1/M guidelines.

This study was completed by a comparison of therapeutic outcomes between HIV-1/O- and HIV-1/M-infected patients to determine if the divergence between groups had an impact on the efficacy of standardized cART [101]. An open nonrandomized clinical trial compared the immuno-virological responses to cART and clinical outcomes; the regimens were based on 2 NRTI + 1 PI/r and were proposed to naïve patients, infected with HIV-1/O (n = 47) and HIV-1/M (n = 94), paired according to criteria. The two regimens were based on 2 NRTI + 1 PI/1. The endpoints were the proportion of patients with undetectable pVL (threshold 60 cp/mL) and the CD4+ count at baseline and W24, W48 and W96. Data showed for the first time a difference of +1 Log in the pVL at baseline for HIV-1/M-infected patients compared to HIV-1/O-infected patients. However, this difference had no impact on reaching undetectable pVL; indeed, no difference was significant at the different times of follow-up. At W96, a virological success >84% was observed for the two groups. Despite a slight increase in CD4+ for HIV-1/M at W48 and W96, there was no significant difference when considering the baseline pVL and inclusion criteria of both groups. Regarding the clinical outcomes (occurrence of events or death), there was no significant difference between the two groups. So, this work demonstrated similar immuno-virological and clinical evolution between the two groups of patients.

These two studies have thus shown that recommendations of therapeutic management for group M-infected patients are adapted for group O infections, and confirmed in vitro data that NNRTIs do not have to be used for these patients. These studies focused on cART mostly based on NRTI + IP/r, so there was also a need to determine the efficacy of a combination based on new drugs such as INSTIs.

A first observational study was conducted on the infected patients of the French RES-O network to investigate the virological outcome for the patients receiving an INSTI-based ART [102]. Nearly 30% of the cohort had an INSTI-based combination during their follow-up, mainly with raltegravir and with a mean duration of 11.9 months. One-third of them had a virological failure at INSTI initiation. However, 90% reached a virological success with undetectable pVL at their most recent visit. Only six patients had a positive pVL (range from 1.7 Log cp/mL to 3.5 Log cp/mL) at the last point of follow-up but had various clinical situations: type of combination (single tablet regimen, dual therapy, etc.), duration of INSTI-based regimen (from 2 to 78 months), previous virological situation (two patients with positive VL 1.8 and 3.9 Log cp/mL). We observed the selection at failure of similar mutations associated with resistance for HIV-1/M (Y143C, Q148R and N155H). We also found mutations present at baseline such as T97A and E17Q. Thus, HIV-1 group O could have a similar pattern of INSTI-resistance mutations to that of HIV-1 group M.

Another observational study specifically focused on the virological response of patients receiving the combination containing bictegravir [103]. We found 6% of the cohort received the combination, with 75% already in virological success from a previous antiretroviral combination. Again, the rate of virological success at the last follow-up was high (87.5%). However, one patient still experienced failure at 3.6 Log cp/mL during the bictegravir regimen. Interestingly no resistance-associated mutation was observed, and undetectability was finally reached 2 months later with the same antiretroviral combination.

**Figure 3:**
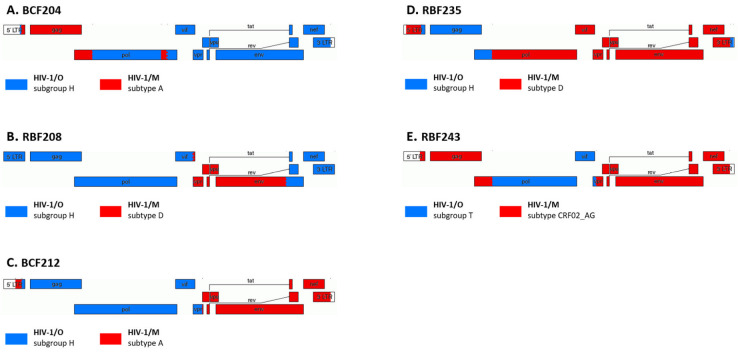
Recombination pattern of five HIV-1/MO recombinant forms. The group origin is indicated in red (group M) and blue (group O). This genomic representation was created using the Recombinant HIV Drawing Tool (http://www.hiv.lanl.gov/content/sequence/DRAW_CRF/recom_mapper.html accessed on 3 february 2023).

As previously indicated in this paper, variants of groups M and O can recombine to create new forms with distinct patterns. These MO recombinant forms have to be managed carefully depending on the therapeutic target. Indeed, since some drugs are inefficient against group O viruses, the therapeutic strategy needs to take into account genome regions belonging to group O. As few patients are infected by these forms, data are very rare. Lastly, for five patients monitored in France, a follow-up could be performed with a median of 35 samples per patient (min = 27; max = 56) and a median of time of 176 months (min = 67; max = 270), between 1999 and 2022. The recombinant patterns are presented in Figure 3.

This unique series allowed obtaining some information on the immuno-virological response for these atypical infections. For four patients (BCF204, RBF208, BCF212 and RBF243), the last point of follow-up showed a cART efficacy with CD4+ between 277 and 731 cells/µL and a pVL undetectable at a threshold of 40 cp/mL (Table 1).

These data demonstrated that an adapted management taking into account the variant leads to an efficient immuno-virological response. For the last one, RBF235, due to undiagnosed HIV-1/M + O coinfection and subsequent emergence of an HIV-1/MO recombinant, and also to adherence difficulties, the last point of follow-up showed virological failure after six distinct regimens (including use of non-recommended NNRTI drugs). Indeed, the patient had 365 CD4+/µL and a pVL at 3.7 Log_10_ copies/mL. In conclusion, there is a need for the genomic profile of these patients to be well known before starting cART and adapting strategies. However, there is no difficulty in managing them using current recommendations based on HIV-1/M therapeutic management, with the exception of NNRTI-based cART if RT belongs to group O.

For HIV-1/N, data are rare. Only three studies have reported response to ART. For two patients, one chronically infected and the other with primary infection, efficacy was observed using ART based on stavudine, lamivudine, and nevirapine [33] and tenofovir, emtricitabine, darunavir-ritonavir, raltegravir, and maraviroc, respectively [23]. The last case corresponded to a 30-year-old infected woman with sustained virological failure after two distinct regimens [77].

For HIV-1/P, the several years of follow-up of the unique infected patient gave original information on natural history during the five years without antiretroviral therapy [41]. The immune response was relatively stable with a mean (min;max) of 326 (240;430) CD4+ cells/μL, despite a consistently high pVL at 4.7 Log cp/mL on average. A drastic reduction in the pVL was observed after three months of antiretroviral therapy (three-drug combination to achieve undetectability). Virological success was also maintained during the nine following years, until the end of the follow-up. A gain in CD4+ was associated with a mean of 648 CD4+ cells/μL during the treatment period.

## 5. Conclusions

HIV-1 non-M strains are known to be divergent genetically. In the past, this led to an important impact on the management of patients infected by such strains. However, due to a better knowledge of their genetic characteristics and susceptibility to drugs, the management has been improved. Viral monitoring is now possible with numerous commercial kits, and it has been shown that several drugs are efficient. This new context, with a very low prevalence of these infections, allows us to be optimistic concerning their elimination by 2030, which could be easier than for the pandemic HIV-1 group M and HIV-2 strains. However, physicians and virologists have to be vigilant due to the persistence of natural resistance to a few drugs in the context of no specific diagnosis or specific detection in most countries.

## Figures and Tables

**Table 1:** Data for the 5 patients infected by an HIV-1/MO with multiple sequential samples.

	Time of Follow-Up	Number of Samples	Year of Last Sample	pVL	CD4+
	(Months)			(Log_10_ cp/mL)	Cells/µL
BCF204	67	35	2012	undetectable	731
RBF208	176	30	2018	undetectable	277
BCF212	139	39	2018	undetectable	689
RBF235	259	56	2021	3.7	365
RBF243	270	27	2022	undetectable	621

## Data Availability

Data are available in the papers cited in the “references” section.

## References

[1] Barré-Sinoussi F., Chermann J.C., Rey F., Nugeyre M.T., Chamaret S., Gruest J., Dauguet C., Axler-Blin C., Vézinet-Brun F., Rouzioux C. (1983). T-lymphotropic retrovirus from a patient at risk for acquired immune deficiency syndrome (AIDS). Science.

[2] Barin F., Denis F., Allan J., M’Boup S., Kanki P., Lee T., Essex M. (1985). Serological evidence for virus related to simian T-lymphotropic retrovirus III in residents of west Africa. Lancet.

[3] Clavel F., Guétard D., Brun-Vézinet F., Chamaret S., Rey M.-A., Santos-Ferreira M.O., Laurent A.G., Dauguet C., Katlama C., Rouzioux C. (1986). Isolation of a new human retrovirus from West African patients with AIDS. Science.

[4] De Leys R., Vanderborght B., Vanden Haesevelde M., Heyndrickx L., van Geel A., Wauters C., Bernaerts R., Saman E., Nijs P., Willems B. (1990). Isolation and partial characterization of an unusual human immunodeficiency retrovirus from two persons of west-central African origin. J. Virol..

[5] Simon F., Mauclère P., Roques P., Loussert-Ajaka I., Müller-Trutwin M., Saragosti S., Georges-Courbot M.C., Barré-Sinoussi F., Brun-Vézinet F. (1998). Identification of a new human immunodeficiency virus type 1 distinct from group M and group O. Nat. Med..

[6] Plantier J.-C., Leoz M., Dickerson J.E., De Oliveira F., Cordonnier F., Lemée V., Damond F., Robertson D.L., Simon F. (2009). A new human immunodeficiency virus derived from gorillas. Nat. Med..

[7] Keele B.F., Van Heuverswyn F., Li Y., Bailes E., Takehisa J., Santiago M.L., Bibollet-Ruche F., Chen Y., Wain L.V., Liegeois F. (2006). Chimpanzee reservoirs of pandemic and nonpandemic HIV-1. Science.

[8] Van Heuverswyn F., Li Y., Bailes E., Neel C., Lafay B., Keele B.F., Shaw K.S., Takehisa J., Kraus M.H., Loul S. (2007). Genetic diversity and phylogeographic clustering of SIVcpzPtt in wild chimpanzees in Cameroon. Virology.

[9] D’Arc M., Ayouba A., Esteban A., Learn G.H., Boué V., Liegeois F., Etienne L., Tagg N., Leendertz F.H., Boesch C. (2015). Origin of the HIV-1 group O epidemic in western lowland gorillas. Proc. Natl. Acad. Sci. USA.

[10] Preston B.D., Poiesz B.J., Loeb L.A. (1988). Fidelity of HIV-1 reverse transcriptase. Science.

[11] Mansky L.M., Temin H.M. (1995). Lower in vivo mutation rate of human immunodeficiency virus type 1 than that predicted from the fidelity of purified reverse transcriptase. J. Virol..

[12] Ho D.D., Neumann A.U., Perelson A.S., Chen W., Leonard J.M., Markowitz M. (1995). Rapid turnover of plasma virions and CD4 lymphocytes in HIV-1 infection. Nature.

[13] Smyth R.P., Davenport M.P., Mak J. (2012). The origin of genetic diversity in HIV-1. Virus Res..

[14] Vijay N.N.V., Vasantika, Ajmani R., Perelson A.S., Dixit N.M. (2008). Recombination increases human immunodeficiency virus fitness, but not necessarily diversity. J. Gen. Virol..

[15] Streeck H., Li B., Poon A., Schneidewind A., Gladden A.D., Power K.A., Daskalakis D., Bazner S., Zuniga R., Brander C. (2008). Immune-driven recombination and loss of control after HIV superinfection. J. Exp. Med..

[16] Nora T., Charpentier C., Tenaillon O., Hoede C., Clavel F., Hance A.J. (2007). Contribution of recombination to the evolution of human immunodeficiency viruses expressing resistance to antiretroviral treatment. J. Virol..

[17] Simon-Loriere E., Holmes E.C. (2011). Why do RNA viruses recombine?. Nat. Rev. Microbiol..

[18] Rambaut A., Posada D., Crandall K.A., Holmes E. (2004). The causes and consequences of HIV evolution. Nat. Rev. Genet..

[19] Hemelaar J., Gouws E., Ghys P.D., Osmanov S. (2006). Global and regional distribution of HIV-1 genetic subtypes and recombinants in 2004. Aids.

[20] Peeters M., Liegeois F., Torimiro N., Bourgeois A., Mpoudi E., Vergne L., Saman E., Delaporte E., Saragosti S. (1999). Characterization of a highly replicative intergroup M/O human immunodeficiency virus type 1 recombinant isolated from a Cameroonian patient. J. Virol..

[21] Takehisa J., Zekeng L., Ido E., Yamaguchi-Kabata Y., Mboudjeka I., Harada Y., Miura T., Kaptué L., Hayami M. (1999). Human immunodeficiency virus type 1 intergroup (M/O) recombination in cameroon. J. Virol..

[22] Mourez T., Simon F., Plantier J.-C. (2013). Non-M Variants of Human Immunodeficiency Virus Type 1. Clin. Microbiol. Rev..

[23] Delaugerre C., De Oliveira F., Lascoux-Combe C., Plantier J.-C., Simon F. (2011). HIV-1 group N: Travelling beyond Cameroon. Lancet.

[24] Delaporte E., Janssens W., Peeters M., Buvé A., Dibanga G., Perret J.-L., Ditsambou V., Mba J.-R., Courbot M.-C.G., Georges A. (1996). Epidemiological and molecular characteristics of HIV infection in Gabon, 1986–1994. Aids.

[25] Zekeng L., Sima J.O., Hampl H., Ndemesogo J.M., Ntutumu J., Sima V., Devare S., Kaptue L., Gürtler L. (1997). Update on HIV-1 group O infection in Equatorial Guinea, Central Africa. Aids.

[26] Hunt J.C., Brennan C.A., Golden A.M., Yamaguchi J., Lund J.K., Vallari A.S., Hickman R.K., Zekeng L., Gürtler L.G., Hampl H. (1997). Molecular analyses of HIV-1 group O and HIV-2 variants from Africa. Leukemia.

[27] Bibollet-Ruche F., Peeters M., Mboup S., Ekaza E., Gandji R., Torimiro J., Mpoudi E.N., Amblard J., Dibanga G., Saidou M. (1998). Molecular characterization of the envelope transmembrane glycoprotein of 13 new human immunodeficiency virus type 1 group O strains from six different African countries. AIDS Res. Hum. Retrovir..

[28] Vessière A., Rousset D., Kfutwah A., Leoz M., Depatureaux A., Simon F., Plantier J.-C. (2010). Diagnosis and monitoring of HIV-1 group O-infected patients in Cameroun. Am. J. Ther..

[29] De Oliveira F., Mourez T., Vessiere A., Ngoupo P.A., Alessandri-Gradt E., Simon F., Rousset D., Plantier J.-C. (2017). Multiple HIV-1/M + HIV-1/O dual infections and new HIV-1/MO inter-group recombinant forms detected in Cameroon. Retrovirology.

[30] Vergne L., Bourgeois A., Mpoudi-Ngole E., Mougnutou R., Mbuagbaw J., Liegeois F., Laurent C., Butel C., Zekeng L., Delaporte E. (2003). Biological and genetic characteristics of HIV infections in Cameroon reveals dual group M and O infections and a correlation between SI-inducing phenotype of the predominant CRF02_AG variant and disease stage. Virology.

[31] Ngoupo P.A., Sadeuh-Mba S.A., De Oliveira F., Ngono V., Ngono L., Tchendjou P., Penlap V., Mourez T., Njouom R., Kfutwah A. (2016). First evidence of transmission of an HIV-1 M/O intergroup recombinant virus. Aids.

[32] Yamaguchi J., Bodelle P., Vallari A.S., Coffey R., McArthur C.P., Schochetman G., Devare S.G., Brennan C.A. (2004). HIV infections in northwestern Cameroon: Identification of HIV type 1 group O and dual HIV type 1 group M and group O infections. AIDS Res. Hum. Retrovir..

[33] Yamaguchi J., Coffey R., Vallari A., Ngansop C., Mbanya D., Ndembi N., Kaptué L., Gürtler L.G., Bodelle P., Schochetman G. (2006). Identification of HIV type 1 group N infections in a husband and wife in Cameroon: Viral genome sequences provide evidence for horizontal transmission. AIDS Res. Hum. Retrovir..

[34] Yamaguchi J., McArthur C.P., Vallari A., Coffey R., Bodelle P., Beyeme M., Schochetman G., Devare S.G., Brennan C.A. (2006). HIV-1 Group N: Evidence of ongoing transmission in Cameroon. AIDS Res. Hum. Retrovir..

[35] Vallari A., Bodelle P., Ngansop C., Makamche F., Ndembi N., Mbanya D., Kaptué L., Gürtler L.G., McArthur C.P., Devare S.G. (2010). Four New HIV-1 Group N Isolates from Cameroon: Prevalence Continues to Be Low. AIDS Res. Hum. Retrovir..

[36] Ayouba A., Souquières S., Njinku B., Martin P.M.V., Müller-Trutwin M.C., Roques P., Barré-Sinoussi F., Mauclère P., Simon F., Nerrienet E. (2000). HIV-1 group N among HIV-1-seropositive individuals in Cameroon. Aids.

[37] Rodgers M., Vallari A., Harris B., Yamaguchi J., Holzmayer V., Forberg K., Berg M., Kenmenge J., Ngansop C., Awazi B. (2017). Identification of rare HIV-1 Group N, HBV AE, and HTLV-3 strains in rural South Cameroon. Virology.

[38] Rodgers M.A., Vallari A.S., Yamaguchi J., Holzmayer V., Harris B., Toure-Kane C., Mboup S., Badreddine S., McArthur C., Ndembi N. (2018). ARCHITECT HIV Combo Ag/Ab and RealTime HIV-1 Assays Detect Diverse HIV Strains in Clinical Specimens. AIDS Res. Hum. Retrovir..

[39] Roques P., Robertson D.L., Souquière S., Apetrei C., Nerrienet E., Barré-Sinoussi F., Müller-Trutwin M., Simon F. (2004). Phylogenetic characteristics of three new HIV-1 N strains and implications for the origin of group N. Aids.

[40] Tagnouokam Ngoupo P.A., Sadeuh-Mba S.A., De Oliveira F., Ngo-Malabo E.T., Ngono L., Plantier J.C., Kfutwah A., Njouom R. (2018). Short Communication: Characterization of a New HIV-1 Group N Isolate Originating from a Cameroonian Patient. AIDS Res. Hum. Retrovir..

[41] Alessandri-Gradt E., De Oliveira F., Leoz M., Lemee V., Robertson D.L., Feyertag F., Ngoupo P.-A., Mauclere P., Simon F., Plantier J.-C. (2018). HIV-1 group P infection: Towards a dead-end infection?. Aids.

[42] Vallari A., Holzmayer V., Harris B., Yamaguchi J., Ngansop C., Makamche F., Mbanya D., Kaptué L., Ndembi N., Gürtler L. (2011). Confirmation of Putative HIV-1 Group P in Cameroon. J. Virol..

[43] Kabeya C.M., Esu-Williams E., Eni E., Peeters M., Saman E., Delaporte E. (1995). Evidence for HIV-1 group O infection in Nigeria. Lancet.

[44] Songok E., Libondo D., Rotich M., Oogo S., Tukei P. (1996). Surveillance for HIV-1 subtypes O and M in Kenya. Lancet.

[45] Heyndrickx L., Alary M., Janssens W., Davo N., van der Groen G. (1996). HIV-1 group O and group M dual infection in Bénin. Lancet.

[46] Peeters M., Gaye A., Mboup S., Badombena W., Bassabi K., Prince-David M., Develoux M., Liegeois F., van der Groen G., Saman E. (1996). Presence of HIV-1 group O infection in West Africa. Aids.

[47] Peeters M., Gueye A., Mboup S., Bibollet-Ruche F., Ekaza E., Mulanga C., Ouedrago R., Gandji R., Mpele P., Dibanga G. (1997). Geographical distribution of HIV-1 group O viruses in Africa. Aids.

[48] Nkengasong J., Sylla-Koko F., Peeters M., Ellenberger D., Sassan-Morokro M., Ekpini R.-A., Msellati P., Greenberg A.E., Combe P., Rayfield M. (1998). HIV-1 group O virus infection in Abidjan, Côte d’lvoire. Aids.

[49] Hampl H., Sawitzky D., Stöffler-Meilicke M., Groh A., Schmitt M., Eberle J., Gürtler L. (1995). First case of HIV-1 subtype 0 infection in Germany. Infection.

[50] Rayfield M.A., Sullivan P., Bandea C.I., Britvan L., Otten R.A., Pau C.P., Pieniazek D., Subbarao S., Simon P., Schable C.A. (1996). HIV-1 group O virus identified for the first time in the United States. Emerg. Infect. Dis..

[51] Soriano V., Gutierrez M., García-Lerma G., Aguilera O., Mas A., Bravo R., Pérez-Labad M.L., Baquero M., González-Lahoz J. (1996). First case of HIV-1 group O infection in Spain. Vox Sang..

[52] Sullivan P.S., Do A.N., Ellenberger D., Pau C.-P., Paul S., Robbins K., Kalish M., Storck C., Schable C.A., Wise H. (2000). Human immunodeficiency virus (HIV) subtype surveillance of African-born persons at risk for group O and group N HIV infections in the United States. J. Infect. Dis..

[53] Gould K. (1997). Infection with HIV-1 group O. AIDS Patient Care STDs.

[54] Depatureaux A., Leoz M., De Oliveira F., Gueudin M., Damond F., Descamps D., Brun-Vézinet F., Lemée V., Simon F., Barin F. (2010). Diagnostic spécifique et prise en charge des infections par un VIH-1 groupe O: Données de RES-O. Médecine Mal. Infect..

[55] Agut H., Rabanel B., Candotti D., Huraux J.-M., Remy G., Tabary T., Ingrand D., Chippaux C., Chamaret S., Dauguet C. (1992). Isolation of atypical HIV-1-related retrovirus from AIDS patient. Lancet.

[56] Plantier J.-C., Lemée V., Dorval I., Gueudin M., Braun J., Hutin P., Ruffault A., Simon F. (2004). HIV-1 group M superinfection in an HIV-1 group O-infected patient. Aids.

[57] Brand D., Beby-Defaux A., Macé M., Brunet S., Moreau A., Godet C., Jais X., Cazein F., Semaille C., Barin F. (2004). First identification of HIV-1 groups M and O dual infections in Europe. Aids.

[58] De Oliveira F., Cappy P., Lemée V., Moisan A., Pronier C., Bocket L., Bouvier-Alias M., Chaix M.-L., Gault E., Morvan O. (2018). Detection of numerous HIV-1/MO recombinants in France. Aids.

[59] Vessière A., Leoz M., Brodard V., Strady C., Lemée V., Depatureaux A., Simon F., Plantier J.-C. (2010). First evidence of a HIV-1 M/O recombinant form circulating outside Cameroon. Aids.

[60] Moisan A., De Oliveira F., Pronier C., Cappy P., Maillard A., Plantier J.C. (2020). In vivo emergence of an HIV-1/MO recombinant revealed undiagnosed HIV-1/M+O co-infection. Clin. Microbiol. Infect..

[61] Moisan A., De Oliveira F., Cappy P., Ngoupo P.A., Njouom R., Plantier J.C. Evolution of HIV-1 groups M and O: Genetic comparative analysis of 23 HIV-1/MO inter-group recombinant forms. Proceedings of the 9th IAS Conference on HIV Science.

[62] Gautheret-Dejean A., Mesmin-Poho S., Birguel J., Lemée V., Huraux J.-M., Plantier J.-C. (2008). Unequal detection of HIV type 1 group O infection by simple rapid tests. Clin. Infect. Dis..

[63] Aghokeng A.F., Mpoudi-Ngole E., Dimodi H., Atem-Tambe A., Tongo M., Butel C., Delaporte E., Peeters M. (2009). Inaccurate diagnosis of HIV-1 group M and O is a key challenge for ongoing universal access to antiretroviral treatment and HIV prevention in Cameroon. PLoS ONE.

[64] Mourez T., Lemée V., Delbos V., Delaugerre C., Alessandri-Gradt E., Etienne M., Simon F., Chaix M.-L., Plantier J.-C. (2018). HIV rapid screening tests and self-tests: Be aware of differences in performance and cautious of vendors. Ebiomedicine.

[65] Zouhair S., Roussin-Bretagne S., Moreau A., Brunet S., Laperche S., Maniez M., Barin F., Harzic M. (2006). Group O Human Immunodeficiency Virus Type 1 Infection That Escaped Detection in Two Immmunoassays. J. Clin. Microbiol..

[66] Plantier J.-C., Djemai M., Lemée V., Reggiani A., Leoz M., Burc L., Vessière A., Rousset D., Poveda J.-D., Henquell C. (2009). Census and Analysis of Persistent False-Negative Results in Serological Diagnosis of Human Immunodeficiency Virus Type 1 Group O Infections. J. Clin. Microbiol..

[67] Swanson P., de Mendoza C., Joshi Y., Golden A., Hodinka R.L., Soriano V., Devare S.G., Hackett J. (2005). Impact of human immunodeficiency virus type 1 (HIV-1) genetic diversity on performance of four commercial viral load assays: LCx HIV RNA Quantitative, AMPLICOR HIV-1 MONITOR v1.5, VERSANT HIV-1 RNA 3.0, and NucliSens HIV-1 QT. J. Clin. Microbiol..

[68] Plantier J.-C., Gueudin M., Damond F., Braun J., Mauclère P., Simon F. (2003). Plasma RNA quantification and HIV-1 divergent strains. Am. J. Ther..

[69] Gueudin M., Plantier J.C., Lemée V., Schmitt M.P., Chartier L., Bourlet T., Ruffault A., Damond F., Vray M., Simon F. (2007). Evaluation of the Roche Cobas TaqMan and Abbott RealTime extraction-quantification systems for HIV-1 subtypes. Am. J. Ther..

[70] Sire J.-M., Vray M., Merzouk M., Plantier J.-C., Pavie J., Maylin S., Timsit J., Lascoux-Combe C., Molina J.-M., Simon F. (2011). Comparative RNA quantification of HIV-1 group M and non-M with the Roche Cobas AmpliPrep/Cobas TaqMan HIV-1 v2.0 and Abbott Real-Time HIV-1 PCR assays. Am. J. Ther..

[71] Tang N., Huang S., Salituro J., Mak W.B., Cloherty G., Johanson J., Li Y.H., Schneider G., Robinson J., Hackett J. (2007). A RealTime HIV-1 viral load assay for automated quantitation of HIV-1 RNA in genetically diverse group M subtypes A-H, group O and group N samples. J. Virol. Methods.

[72] Alessandri-Gradt E., Unal G., Baron A., Leoz M., Gueudin M., Plantier J.-C., Network T.R.-O. (2021). Performance Analysis of Three Commercial Kits Designed for RNA Quantification of HIV-1 Group O Variants. Am. J. Ther..

[73] Berger A., Muenchhoff M., Hourfar K., Kortenbusch M., Ambiel I., Stegmann L., Heim A., Sarrazin C., Ehret R., Daniel V. (2020). Severe underquantification of HIV-1 group O isolates by major commercial PCR-based assays. Clin. Microbiol. Infect..

[74] Gueudin M., Baron A., Alessandri-Gradt E., Lemée V., Mourez T., Etienne M., Plantier J.-C. (2016). Performance Evaluation of the New HIV-1 Quantification Assay, Xpert HIV-1 Viral Load, on a Wide Panel of HIV-1 Variants. Am. J. Ther..

[75] Mourez T., Delaugerre C., Vray M., Lemée V., Simon F., Plantier J.C. (2015). Comparison of the bioMérieux NucliSENS EasyQ HIV-1 v2.0-HIV-1 RNA quantification assay versus Abbott RealTime HIV-1 and Roche Cobas TaqMan HIV-1 v2.0 on current epidemic HIV-1 variants. J. Clin. Virol..

[76] Depatureaux A., Charpentier C., Leoz M., Unal G., Damond F., Kfutwah A., Vessière A., Simon F., Plantier J.-C. (2011). Impact of HIV-1 group O genetic diversity on genotypic resistance interpretation by algorithms designed for HIV-1 group M. Am. J. Ther..

[77] Péré H., Roques P., Talla F., Meillo H., Charpentier C., Bélec L. (2015). Sustained virological failure in Cameroonese patient infected by HIV-1 group N evidenced by sequence-based genotyping assay. Aids.

[78] Descamps D., Collin G., Letourneur F., Apetrei C., Damond F., Loussert-Ajaka I., Simon F., Saragosti S., Brun-Vézinet F. (1997). Susceptibility of human immunodeficiency virus type 1 group O isolates to antiretroviral agents: In vitro phenotypic and genotypic analyses. J. Virol..

[79] Tebit D.M., Patel H., Ratcliff A., Alessandri E., Liu J., Carpenter C., Plantier J.-C., Arts E.J. (2016). HIV-1 Group O Genotypes and Phenotypes: Relationship to Fitness and Susceptibility to Antiretroviral Drugs. AIDS Res. Hum. Retrovir..

[80] Parkin N.T., Schapiro J.M. (2004). Antiretroviral drug resistance in non-subtype B HIV-1, HIV-2 and SIV. Antivir. Ther..

[81] Tuaillon E., Gueudin M., Lemée V., Gueit I., Roques P., Corrigan G.E., Plantier J.-C., Simon F., Braun J. (2004). Phenotypic Susceptibility to Nonnucleoside Inhibitors of Virion-Associated Reverse Transcriptase From Different HIV Types and Groups. Am. J. Ther..

[82] Villabona-Arenas C.J., Domyeum J., Mouacha F., Butel C., Delaporte E., Peeters M., Mpoudi-Ngole E., Aghokeng A.F. (2015). HIV-1 group O infection in Cameroon from 2006 to 2013: Prevalence, genetic diversity, evolution and public health challenges. Infect. Genet. Evol..

[83] Tebit D.M., Lobritz M., Lalonde M., Immonen T., Singh K., Sarafianos S., Herchenröder O., Kräusslich H.-G., Arts E.J. (2010). Divergent Evolution in Reverse Transcriptase (RT) of HIV-1 Group O and M Lineages: Impact on Structure, Fitness, and Sensitivity to Nonnucleoside RT Inhibitors. J. Virol..

[84] Leoz M., Feyertag F., Kfutwah A., Mauclère P., Lachenal G., Damond F., De Oliveira F., Lemée V., Simon F., Robertson D.L. (2015). The Two-Phase Emergence of Non Pandemic HIV-1 Group O in Cameroon. PLOS Pathog..

[85] de Baar M.P., Janssens W., de Ronde A., Fransen K., Colebunders R., Kestens L., van der Groen G., Goudsmit J. (2000). Natural residues versus antiretroviral drug-selected mutations in HIV type 1 group O reverse transcriptase and protease related to virological drug failure in vivo. AIDS Res. Hum. Retrovir..

[86] Luk K.-C., Kaptue L., Zekeng L., Soriano V., Gürtler L., Devare S.G., Schochetman G., Hackett J. (2001). Naturally occurring sequence polymorphisms within HIV type 1 group O protease. AIDS Res. Hum. Retrovir..

[87] Depatureaux A., Charpentier C., Collin G., Leoz M., Descamps D., Vessière A., Damond F., Rousset D., Brun-Vézinet F., Plantier J.-C. (2010). Baseline Genotypic and Phenotypic Susceptibilities of HIV-1 Group O to Enfuvirtide. Antimicrob. Agents Chemother..

[88] Roques P., Robertson D., Souquière S., Damond F., Ayouba A., Farfara I., Depienne C., Nerrienet E., Dormont D., Vézinetd F. (2002). Phylogenetic Analysis of 49 Newly Derived HIV-1 Group O Strains: High Viral Diversity but No Group M-like Subtype Structure. Virology.

[89] Yamaguchi J., Vallari A.S., Swanson P., Bodelle P., Kaptué L., Ngansop C., Zekeng L., Gürtler L.G., Devare S.G., Brennan C.A. (2002). Evaluation of HIV type 1 group O isolates: Identification of five phylogenetic clusters. AIDS Res. Hum. Retrovir..

[90] Alessandri-Gradt E., Charpentier C., Leoz M., Mourez T., Descamps D., Plantier J.-C. (2018). Impact of natural polymorphisms of HIV-1 non-group M on genotypic susceptibility to the attachment inhibitor fostemsavir. J. Antimicrob. Chemother..

[91] Leoz M., Depatureaux A., Vessière A., Roquebert B., Damond F., Rousset D., Roques P., Simon F., Plantier J.-C. (2008). Integrase polymorphism and HIV-1 group O diversity. Aids.

[92] Briz V., Garrido C., Poveda E., Morello J., Barreiro P., de Mendoza C., Soriano V. (2009). Raltegravir and etravirine are active against HIV type 1 group O. AIDS Res. Hum. Retrovir..

[93] Alessandri-Gradt E., Collin G., Tourneroche A., Bertine M., Leoz M., Charpentier C., Unal G., Descamps D., Plantier J.C. (2017). HIV-1 non-group M phenotypic susceptibility to integrase strand transfer inhibitors. J. Antimicrob. Chemother..

[94] Martin C., Gracias S., Charpentier C., Descamps D., Le Hingrat Q., Plantier J.-C., Alessandri-Gradt E. (2021). HIV-1 non-group M phenotypic susceptibility in vitro to bictegravir and cabotegravir. J. Antimicrob. Chemother..

[95] Tsiang M., Jones G.S., Goldsmith J., Mulato A., Hansen D., Kan E., Tsai L., Bam R.A., Stepan G., Stray K.M. (2016). Antiviral Activity of Bictegravir (GS-9883), a Novel Potent HIV-1 Integrase Strand Transfer Inhibitor with an Improved Resistance Profile. Antimicrob. Agents Chemother..

[96] Depatureaux A.M., Leoz M.M., Le Moal G., Pathé J.-P., Pavie J., Batisse D., Daneluzzi V., Genet P.M., Gerard L., Lascaux-Cametz A.-S. (2012). Raltegravir-Based Regimens Are Effective in HIV-1 Group O–Infected Patients. Am. J. Ther..

[97] Rodes B., De Mendoza C., Rodgers M., Newell A., Jimenez V., Lopez-Brugada R.M., Soriano V. (2005). Treatment response and drug resistance in patients infected with HIV type 1 group O viruses. AIDS Res. Hum. Retrovir..

[98] Aghokeng A.F., Kouanfack C., Peeters M., Mpoudi-Ngole E., Delaporte E. (2013). Successful integrase inhibitor-based highly active antiretroviral therapy for a multidrug-class-resistant HIV type 1 group O-infected patient in Cameroon. AIDS Res. Hum. Retrovir..

[99] Poveda E., Barreiro P., Rodés B., Soriano V. (2005). Enfuvirtide is active against HIV type 1 group O. AIDS Res. Hum. Retrovir..

[100] Unal G., Alessandri-Gradt E., Leoz M., Pavie J., Lefèvre C., Panjo H., Charpentier C., Descamps D., Barin F., Simon F. (2017). Human Immunodeficiency Virus Type 1 Group O Infection in France: Clinical Features and Immunovirological Response to Antiretrovirals. Clin. Infect. Dis..

[101] Kouanfack C., Unal G., Schaeffer L., Kfutwah A., Aghokeng A., Mougnutou R., Tchemgui-Noumsi N., Alessandri-Gradt E., Delaporte E., Simon F. (2019). Comparative Immunovirological and Clinical Responses to Antiretroviral Therapy Between HIV-1 Group O and HIV-1 Group M Infected Patients. Clin. Infect. Dis..

[102] Alessandri-Gradt E., Unal G., Leoz M., Plantier J.-C. (2019). Virological response to integrase strand transfer inhibitor-based antiretroviral combinations in HIV-1 group O-infected patients. Aids.

[103] Martin C., Unal G., Plantier J.C., Alessandri-Gradt E. (2022). Bictegravir-based antiretroviral therapy in HIV-1 group O patients: Data from real-life bictegravir/emtricitabine/tenofovir alafenamide switches. J. Antimicrob. Chemother..

